# Supplementary material for: ProteinShader: illustrative rendering of macromolecules
Source: BMC Struct Biol. 2009 Mar 30;9:19. doi: 10.1186/1472-6807-9-19 (PMC2672931; doi:10.1186/1472-6807-9-19)
Supplement: Additional file 1 — ProteinShader program without source code. This compressed file contains the complete ProteinShader program including associated libraries, but no source code. A README.txt file gives an overview of the ProteinShader distribution, and the index.html file in the help subdirectory has directions on getting started with the program as well as a set of tutorials. [file 1472-6807-9-19-S1.zip › ProteinShader-beta-0_9_4-binary/help/api/org/proteinshader/graphics/ExtrudedShape.html]

ExtrudedShape (ProteinShader API)


|  |  |  |
| --- | --- | --- |
| **Overview** \| **Package** \| **Class** \| **Use** \| **Tree** \| **Deprecated** \| **Index** \| **Help** | |  |
| **PREV CLASS**   **NEXT CLASS** | **FRAMES**    **NO FRAMES**     **All Classes** |  |
| SUMMARY: NESTED \| FIELD \| CONSTR \| METHOD | DETAIL: FIELD \| CONSTR \| METHOD |  |


---


## org.proteinshader.graphics Class ExtrudedShape

```
java.lang.Object
  org.proteinshader.graphics.Shape
      org.proteinshader.graphics.ExtrudedShape
```

**Direct Known Subclasses:**: FrenetFrames, Ribbon, Tube

---

``` public abstract class ExtrudedShape extends Shape ```

Contains common methods needed by classes that draw segments of a
three-dimensional tube by sweeping a waist polygon along a spline.

---

| **Field Summary** | |
| --- | --- |
| `static float[]` | `END_CAP_COLOR`             Segment end (carboxyl end) caps are colored red because that is the CPK color for oxygen. |
| `static float[]` | `START_CAP_COLOR`             Segment start (amino end) caps are colored light blue because that is the CPK color for nitrogen. |

**Fields inherited from class org.proteinshader.graphics.Shape**

`HALF_PI, PI, SPECULAR_COLOR, TWO_PI`


**Constructor Summary**

`ExtrudedShape()`
  
          Constructor for use by subclasses.


| **Method Summary** | |
| --- | --- |
| `void` | `applyEndCapColor(GL gl, float alpha, float specularExp)`             Calls the setMaterial() method of superclass Shape with the END\_CAP\_COLOR along with the alpha value and specular exponent given as aguments. |
| `void` | `applyStartCapColor(GL gl, float alpha, float specularExp)`             Calls the setMaterial() method of superclass Shape with the START\_CAP\_COLOR along with the alpha value and specular exponent given as aguments. |
| `int` | `createDisplayList(GL gl, LocalFrame[] frames)`             Uses an array of LocalFrame objects to draw a segment of a tube and saves the geometry (a collection of vertices) as an OpenGL display list. |
| `void` | `createDisplayLists(GL gl, LocalFrame[] frames, SegmentListInfo info)`             Creates three OpenGL display lists: one created by calling draw(), one created by calling drawStartCap(), and one created by calling drawEndCap(). |
| `int` | `createEndCapDisplayList(GL gl, LocalFrame[] frames)`             Uses the last LocalFrame in the array to position a segment end cap that is saved as an OpenGL display list. |
| `int` | `createStartCapDisplayList(GL gl, LocalFrame[] frames)`             Uses the first LocalFrame in the array to position a segment start cap that is saved as an OpenGL display list. |
| `abstract  void` | `draw(GL gl, LocalFrame[] frames)`             Uses an array of LocalFrame objects to draw a segment of an extruded shape. |
| `abstract  void` | `drawEndCap(GL gl, LocalFrame frame)`             Creates a cap for the end of a segment by drawing the waist polygon. |
| `abstract  void` | `drawStartCap(GL gl, LocalFrame frame)`             Creates a cap for the beginning of a segment by drawing the waist polygon. |

**Methods inherited from class org.proteinshader.graphics.Shape**

`deleteDisplayList, executeDisplayList, executeDisplayList, setMaterial`

**Methods inherited from class java.lang.Object**

`clone, equals, finalize, getClass, hashCode, notify, notifyAll, toString, wait, wait, wait`

**Field Detail**

### START\_CAP\_COLOR

```
public static final float[] START_CAP_COLOR
```

:   Segment start (amino end) caps are colored light blue
    because that is the CPK color for nitrogen.

---


### END\_CAP\_COLOR

```
public static final float[] END_CAP_COLOR
```

:   Segment end (carboxyl end) caps are colored red because
    that is the CPK color for oxygen.


**Constructor Detail**

### ExtrudedShape

```
public ExtrudedShape()
```

:   Constructor for use by subclasses.


**Method Detail**

### draw

```
public abstract void draw(GL gl,
                          LocalFrame[] frames)
```

:   Uses an array of LocalFrame objects to draw a segment of an
    extruded shape.

    :   **Parameters:**: `gl` - the current GL object.: `frames` - array of LocalFrames for positioning a waist polygon.

---


### drawStartCap

```
public abstract void drawStartCap(GL gl,
                                  LocalFrame frame)
```

:   Creates a cap for the beginning of a segment by drawing the
    waist polygon. The surface normal will be the negative z-axis
    (the tangent) of the local coordinate frame.

    :   **Parameters:**: `gl` - the current GL object.: `frame` - the frame for positioning the waist polygon.

---


### drawEndCap

```
public abstract void drawEndCap(GL gl,
                                LocalFrame frame)
```

:   Creates a cap for the end of a segment by drawing the waist
    polygon. The surface normal will be the negative z-axis (the
    tangent) of the local coordinate frame.

    :   **Parameters:**: `gl` - the current GL object.: `frame` - the frame for positioning the waist polygon.

---


### applyStartCapColor

```
public void applyStartCapColor(GL gl,
                               float alpha,
                               float specularExp)
```

:   Calls the setMaterial() method of superclass Shape with the
    START\_CAP\_COLOR along with the alpha value and specular exponent
    given as aguments.

    :   **Parameters:**: `gl` - the current GL object.: `alpha` - component of RGBA color: `specularExp` - the specular exponent for Phong lighting calculations.

---


### applyEndCapColor

```
public void applyEndCapColor(GL gl,
                             float alpha,
                             float specularExp)
```

:   Calls the setMaterial() method of superclass Shape with the
    END\_CAP\_COLOR along with the alpha value and specular exponent
    given as aguments.

    :   **Parameters:**: `gl` - the current GL object.: `alpha` - component of RGBA color: `specularExp` - the specular exponent for Phong lighting calculations.

---


### createDisplayLists

```
public void createDisplayLists(GL gl,
                               LocalFrame[] frames,
                               SegmentListInfo info)
```

:   Creates three OpenGL display lists: one created by calling draw(),
    one created by calling drawStartCap(), and one created by calling
    drawEndCap(). The start and end caps are saved as separate OpenGL
    display lists so that they can be colored differently than the
    main part of the Segment (which will be a tube or ribbon depending
    on the subclass).

    :   **Parameters:**: `gl` - the current GL object.: `frames` - array of LocalFrames for positioning a waist polygon.: `info` - the info object to store the name (an integer) of each OpenGL display list that is created.

---


### createDisplayList

```
public int createDisplayList(GL gl,
                             LocalFrame[] frames)
```

:   Uses an array of LocalFrame objects to draw a segment of a tube
    and saves the geometry (a collection of vertices) as an OpenGL
    display list.

---


### createStartCapDisplayList

```
public int createStartCapDisplayList(GL gl,
                                     LocalFrame[] frames)
```

:   Uses the first LocalFrame in the array to position a segment start
    cap that is saved as an OpenGL display list.

---


### createEndCapDisplayList

```
public int createEndCapDisplayList(GL gl,
                                   LocalFrame[] frames)
```

:   Uses the last LocalFrame in the array to position a segment end
    cap that is saved as an OpenGL display list.


---


|  |  |  |
| --- | --- | --- |
| **Overview** \| **Package** \| **Class** \| **Use** \| **Tree** \| **Deprecated** \| **Index** \| **Help** | |  |
| **PREV CLASS**   **NEXT CLASS** | **FRAMES**    **NO FRAMES**     **All Classes** |  |
| SUMMARY: NESTED \| FIELD \| CONSTR \| METHOD | DETAIL: FIELD \| CONSTR \| METHOD |  |


---

# *Copyright © 2007-2008*
